# Retrospective Evaluation of the Impact of *SLCO1B1* Variation on Statin Effectiveness

**DOI:** 10.3390/jpm15110511

**Published:** 2025-10-29

**Authors:** Mayeesha Ahmed Feldman, Kendall Billman, Mounia Sennoun, Gloria Ng, Mariam Hussain, Elizabeth G. Schlosser, Ana L. Hincapie, Josiah D. Allen

**Affiliations:** 1 Department of Pharmacy, St. Elizabeth Healthcare, Edgewood, KY 41017, USA; 2 Department of Precision Medicine and Genomic Health, St. Elizabeth Healthcare, Edgewood, KY 41017, USA; kendall.billman@stelizabeth.com (K.B.); josiah.allen@stelizabeth.com (J.D.A.); 3 James L. Winkle College of Pharmacy, University of Cincinnati, Cincinnati, OH 45267, USA; sennouma@mail.uc.edu (M.S.); nggj@mail.uc.edu (G.N.); schloseg@ucmail.uc.edu (E.G.S.); hincapaa@ucmail.uc.edu (A.L.H.); 4 College of Health Professions, Manchester University, Fort Wayne, IN 46845, USA; mhussain2025@manchester.edu; 5 Ambulatory Care Pharmacy, St. Elizabeth Physicians, Erlanger, KY 41018, USA

**Keywords:** SLCO1B1, statin, pharmacogenomics, pharmacogenetics, precision medicine, SAMS, statin-associated musculoskeletal symptoms

## Abstract

**Background**: Solute carrier organic anion transporter family member 1B1 (SLCO1B1) mediates statin uptake into hepatocytes, the primary sites of cholesterol production. While the impact of *SLCO1B1* variation on statin-associated muscle symptoms (SAMS) is well-documented, its role in LDL-C reduction remains understudied. This single-center, retrospective cohort study evaluated whether *SLCO1B1* variation affects statin effectiveness in 213 adults. **Methods**: The *SLCO1B1* variant rs4149056 (NM_006446.5:c.521T>C) was tested to categorize patients by their *SLCO1B1* function: normal, decreased, or poor. The primary endpoint was percent change in LDL-C from baseline to follow-up (≥6 weeks post-statin initiation), with secondary endpoints of SAMS occurrence and statin adherence. **Results**: Overall, the average LDL-C decreased by 32% across all groups. No significant difference in LDL-C reduction was observed between *SLCO1B1* phenotypes (*p* = 0.24). **Conclusions**: *SLCO1B1* variation did not significantly affect LDL-C reduction, SAMS occurrence, or statin adherence. However, the retrospective design and limited adherence data in this study represent important limitations warranting prospective validation studies.

## 1. Introduction

Hydroxymethylglutaryl-coenzyme A (HMG-CoA) reductase inhibitors, more commonly known as statins, are a cornerstone of therapy in the management of hyperlipidemia and cardiovascular risk reduction [1]. Statins treat dyslipidemia by inhibiting hepatic cholesterol synthesis, which in turn causes up-regulation of LDL-C receptors on the cell surface of hepatocytes, thus increasing removal of LDL-C from the bloodstream [2].

Solute carrier organic anion transporter family member 1B1 (SLCO1B1) is a transporter that facilitates the hepatic uptake of statins into the liver [3]. Decreased function of this transporter, inherited through genetic variability, or altered statin exposure due to drug interactions in individuals who phenotypically have decreased or poor *SLCO1B1* function, results in increased systemic exposure to statins, thus increasing the risk of SAMS. SAMS frequently leads to statin discontinuation, leading to higher cholesterol levels and increased cardiovascular risk [1]. The association between *SLCO1B1* variation and SAMS risk is well studied [4,5,6,7,8], and the 2022 Clinical Pharmacogenetics Implementation Consortium (CPIC) guideline for statins [2] provides therapeutic recommendations for statins based on *SLCO1B1* function to minimize SAMS risk with the goal of improving the overall safety, adherence, and effectiveness of statin therapy.

Most studies focus on the association of *SLCO1B1* phenotype with the risk of SAMS. There is a paucity of data on the effect of *SLCO1B1* function on statin effectiveness. Given that most of the body’s cholesterol is produced in the liver [9], decreased function of the SLCO1B1 transporter is expected to decrease the amount of drug reaching the site of action, potentially decreasing its lipid-lowering effect. A small, prospective cohort study by Zakria et al. on hyperlipidemic patients prescribed rosuvastatin 10 mg/day who were followed for 24 weeks showed differential lipid-lowering responses and musculoskeletal symptoms based on *SLCO1B1* common polymorphisms [10]. A study by Sivkov et al. on 180 hyperlipidemic patients treated with rosuvastatin, atorvastatin, or simvastatin found that LDL-C reductions were less pronounced in patients with *SLCO1B1* c.521T>C CC genotype vs. TC or TT genotypes [11]. A meta-analysis of 21 studies by Nguyen et al. found a significant association between LDL-C lowering effectiveness and *SLCO1B1* variants, particularly rs4149056 (c.521T>C), rs2306283 (c.388A>G) and rs11045819 (c.463C>A) polymorphisms [12]. However, these studies are limited by several factors, including a limited number of statins and doses evaluated, small sample size, and study heterogeneity arising from varied statin dosages and treatment durations in the studies included in the meta-analysis by Nguyen et al. [12], which may have confounded the observed associations between *SLCO1B1* polymorphisms and statin effectiveness.

Elevated LDL-C levels are an important risk factor for the development of cardiovascular disease (CVD). The Cholesterol Treatment Trialists (CTT) Collaborators found that an LDL-C reduction of 1 mmol/L was associated with a 21% reduction in major vascular events [13]. Validating associations between *SLCO1B1* function and degree of LDL-C lowering can help guide optimal prescribing of lipid-lowering medications. If *SLCO1B1* function is found to serve as a marker of reduced statin effectiveness, it could be used clinically, by itself or with other patient factors, to triage patients to other lipid-lowering therapies such as proprotein convertase subtilisin/kexin type 9 inhibitors (PCSK9i), resulting in better patient outcomes and potentially better insurance coverage of both pharmacogenomic testing and PCSK9i therapy. The authors therefore sought to assess the effect of *SLCO1B1* variation on statin effectiveness within their patient population, with the primary endpoint being the change in LDL-C and secondary endpoints including occurrence of SAMS and statin adherence.

## 2. Materials and Methods

This single-center cohort study evaluated adult patients treated at a large community health system from January 2018 to August 2024 based on a retrospective chart review. At this site, patients are tested using an external laboratory for a variety of conditions, most commonly mental health, pre-emptive *DPYD* screening, statin intolerance, and appropriate clopidogrel use. The pharmacogenomics program is pharmacist-led, with a weekly pharmacogenomics clinic offering both pre- and post-test consultations, with most testing ordered through physicians. Charts of patients who received *SLCO1B1* testing as part of the 27-gene panel pharmacogenomic (PGx) testing for various indications and were on statin therapy were reviewed (n = 2015).

**Figure 1:**
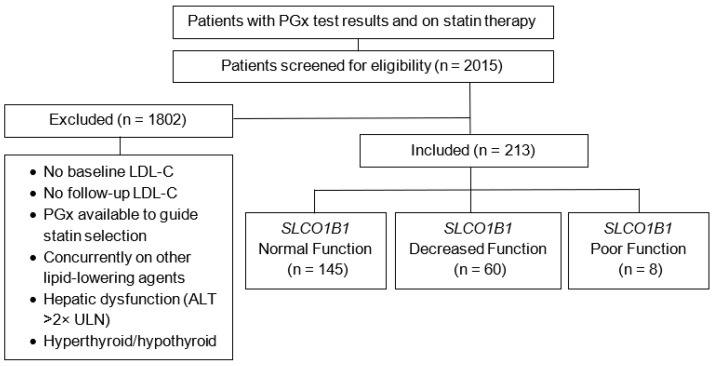
Flowchart of the patient screening and inclusion process. n = number of patients per group; LDL-C = low-density lipoprotein cholesterol; ALT = alanine transaminase level; ULN = upper limit of normal.

Patients were excluded if they were <18 years of age, not prescribed statin therapy, had PGx testing ordered specifically to guide statin selection (including those with a history of statin failure or who had statin therapy initiated after PGx results), were on other lipid-lowering agents during the study period (ezetimibe, PCSK9i, bempedoic acid, gemfibrozil, niacin, or fish oil), or had evidence of thyroid dysregulation (thyroid stimulating hormone level outside of the reference range of 0.4–4.0 mcIU/mL) or hepatic dysfunction (alanine transaminase level > 2× upper limit of normal, i.e., ALT > 58 U/L for males and ALT > 38 U/L for females) during the study period (Figure 1). The tested *SLCO1B1* variant was rs4149056 (NM_006446.5:c.521T>C). The rs4149056 variant (c.521T>C) in the *SLCO1B1* gene is designated as the *5 allele according to PharmVar [14] nomenclature. This variant affects SLCO1B1 transporter activity, with the C allele (*5) associated with reduced protein function compared to the wild-type T allele (*1). The genotype–phenotype relationships are as follows [3]:

- TT genotype (*1/*1): Normal function phenotype
- TC genotype (*1/*5): Decreased function phenotype
- CC genotype (*5/*5): Poor function phenotype

The index date was defined as the date of the baseline LDL-C level obtained within 1 year prior to statin initiation, and the study period was defined as the timeframe between the index date and the date of the follow-up LDL-C level obtained at least 6 weeks after statin initiation. Chart review and data extraction were performed by five reviewers using clearly defined data collection criteria. Any discrepancies identified during the review process were discussed among all reviewers and resolved through group consensus. Additional variables recorded included age, race, sex, body mass index (BMI), smoking status, diabetes, chronic kidney disease (defined as estimated glomerular filtration rate (eGFR) < 60 or chronic kidney disease (CKD) in the problem list), concurrent use of strong or moderate *CYP3A4* inducers and inhibitors (strong inducers included carbamazepine, enzalutamide, and/or rifampin; moderate inducers included dabrafenib, efavirenz, elagolix, eslicarbazepine, lorlatinib, oxcarbazepine, phenobarbital, phenytoin, and/or St. John’s wort; strong inhibitors included ceritinib, clarithromycin, delaviridine, idelalisib, indinavir, itraconazole, ketoconazole, mibefradil, nefazodone, nelfinavir, ribociclib, ritonavir, saquinavir, telaprevir, telithromycin, tucatinib, and/or voriconazole; moderate inhibitors included aprepitant, ciprofloxacin, crizotinib, diltiazem, erythromycin, fluconazole, imatinib, letermovir, netupitant, and/or verapamil) [15], statin medication and dose prescribed, and indication for statin therapy (i.e., primary atherosclerotic cardiovascular disease (ASCVD) prevention or secondary ASCVD prevention, with secondary ASCVD prevention defined as history of a prior ASCVD event, which includes stroke, transient ischemic attack, documented coronary artery disease with stable angina, acute coronary syndromes, coronary or other arterial revascularization, peripheral vascular disease, or aortic aneurysm). CYP3A4*1A = NM_017460.6:c.-392G>A (rs2740574), CYP3A4*22 = NM_017460.6:c.522-191C>T (rs35599367) and CYP3A5*3 = NM_000777.5:c.219-237A>G (rs776746), CYP3A5*6 = NM_000777.5:c.624G>A (rs10264272), CYP3A5*7 = NM_000777.5:c.1035_1036insT (rs41303343) genetic variants as defined by PharmVar nomenclature [14] were also assessed. Although *CYP3A4* is not a CPIC gene, it is the primary metabolizing enzyme for atorvastatin and simvastatin [16], and was therefore assessed as an exploratory endpoint. The primary endpoint was the percent change in LDL-C from index to follow-up, with secondary endpoints of SAMS occurrence and statin adherence.

**Figure 2:**
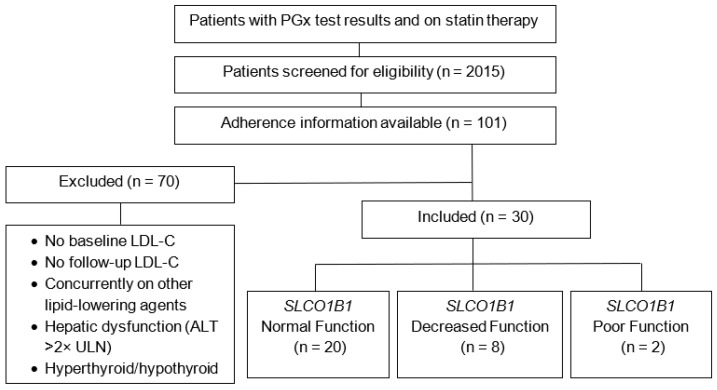
Flowchart of the patient screening and modified inclusion process for adherence. n = number of patients per group; LDL-C = low-density lipoprotein cholesterol; ALT = alanine transaminase level; ULN = upper limit of normal.

SAMS was defined as any myopathy during statin therapy and identified through a stepwise process: 1. Documentation of statin intolerance in the allergies section. 2. Chart documentation of “myopathy” and included only if it was documented to be statin-related. Statin adherence was defined as the proportion of days covered (PDC) of 80% or higher during the study period. For both SAMS and adherence endpoints, patients with missing data were excluded from the analysis of that specific outcome. Due to the small sample size with available adherence data, the exclusion criteria were modified for the adherence endpoint to include patients even if their PGx data were available prior to prescribing a statin (Figure 2). Data analysis was performed with Microsoft^®^ Excel^®^ Version 2503 (Microsoft Corporation, Redmond, WA, USA) and JMP^®^ Pro 17.2.0 (Statistical Discovery LLC, Cary, NC, USA). Baseline characteristics were analyzed using descriptive statistics, except for mean baseline LDL-C by *SLCO1B1* function, which was analyzed using one-way ANOVA. One-way ANOVA was used for the primary outcome and linear regression was used to adjust for confounders. Secondary outcomes of SAMS occurrence and statin adherence were analyzed using chi-square test of independence and Fisher’s exact test, respectively, with logistic regression used to adjust for confounders. Confounders included *CYP3A4* and *CYP3A5* variation, BMI, smoking status, diabetes, CKD, and race.

## 3. Results

A total of 2015 patients were identified as having pharmacogenomic test results who were also prescribed statin therapy during the study period. During the review, 1802 patients were excluded due to the following reasons: no baseline LDL-C, no follow-up LDL-C, PGx results were available to guide statin therapy, concurrent use of other lipid-lowering agents, hepatic dysfunction, and thyroid dysfunction, resulting in 213 patients being included (Figure 1).

**Figure 3:**
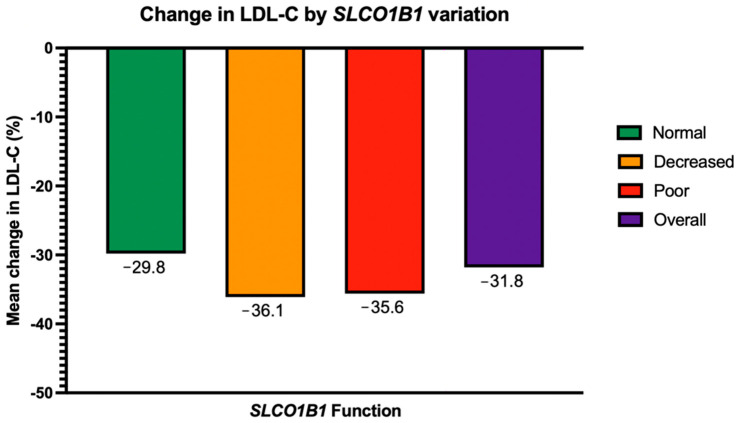
Primary outcome: change in LDL-C by *SLCO1B1* variation. *SLCO1B1* variation is defined as *SLCO1B1* function: normal, decreased, or poor. LDL-C = low-density lipoprotein cholesterol.

Baseline characteristics are summarized in Table 1. The average duration of statin therapy at the time of the first follow-up LDL-C level was 10 months. The mean overall baseline LDL-C was 142 mg/dL (±35.8 mg/dL). The mean baseline LDL-C was not significantly different between the 3 *SLCO1B1* function groups (141 mg/dL, 141 mg/dL and 153 mg/dL for normal, decreased, and poor *SLCO1B1* function, respectively, *p* = 0.65). Overall, 68% of the patients were predicted to have normal *SLCO1B1* function, 28% decreased function, and 4% poor function. LDL-C decreased by an average of 31.8% across all 3 *SLCO1B1* groups. LDL-C decreased on average by 29.8% (95% CI: −33.9, −25.7), 36.1% (95% CI: −42.5, −29.7), and 35.6% (95% CI: −53.0, −18.2) in the normal, decreased, and poor *SLCO1B1* function groups, respectively (Figure 3), and this difference across the groups was not found to be statistically significant (*p* = 0.24, Table 2). Adjusting for *CYP3A4* and *CYP3A5* variation, BMI, smoking status, diabetes, CKD, and race did not significantly impact this finding. In a sub-group analysis, adjusting for statin intensity did not affect changes in LDL-C based on *SLCO1B1* variation (Table 2).

Overall, SAMS occurred in 29 patients and *SLCO1B1* variation did not significantly impact SAMS occurrence (*p* = 0.24, Table 3). Statin adherence data was available for 101 patients out of the total 2015 patients screened. Based on the modified exclusion criteria for this endpoint as described in Section 2, 30 patients were included for analysis of adherence to statin therapy. In this sample, 22 patients were adherent to statin therapy based on our adherence cutoff of 80% PDC or higher, and *SLCO1B1* variation was not found to significantly impact statin adherence (*p* = 1.00, Table 4). A subgroup analysis exploring the effect of SAMS on changes in LDL-C and statin adherence found that the decrease in LDL-C was significantly greater in patients who did not have SAMS (0.04, Table 5); however, SAMS did not significantly affect adherence in this sample (*p* = 0.44, Table 6).

## 4. Discussion

This single-center retrospective cohort study examined the relationship between *SLCO1B1* genetic variation and statin effectiveness, SAMS occurrence, and adherence in a real-world clinical setting. Contrary to the authors’ hypothesis and previous studies, the findings do not support a significant impact of *SLCO1B1* phenotype on any of these clinical outcomes.

This study contributes to the limited body of literature evaluating the impact of *SLCO1B1* variation on statin effectiveness. Unlike previous research that has predominantly focused on the well-established association between *SLCO1B1* and SAMS risk [4,5,6,7,8], this investigation specifically evaluated lipid-lowering effectiveness across different *SLCO1B1* phenotypes. The inclusion of all currently available statins at varying intensities (high, moderate, and low) provides a comprehensive assessment of statin response patterns that may be generalizable to diverse clinical scenarios. Additionally, the attempt to incorporate third party fill history as a surrogate for adherence represents a key methodological consideration that is often lacking in retrospective chart review studies, as adherence significantly impacts both effectiveness and safety outcomes.

The assessment of *CYP3A4* and *CYP3A5* metabolizer status and drug interactions with strong and moderate *CYP3A4* inducers and inhibitors further strengthens the analysis by accounting for potential confounding effects of drug metabolism on statin exposure and response. As only 4 out of the 213 included patients were on an interacting *CYP3A4* inducer/inhibitor, this confounder was excluded from the analysis.

Several non-genetic factors also play a role in statin pharmacokinetics and may influence therapeutic outcomes of these agents. To address this, this study also analyzed the impact of race, BMI, smoking, diabetes, CKD, and excluded confounders such as concurrent use of other lipid-lowering agents, hepatic dysfunction, and thyroid dysfunction.

The subgroup analysis finding that patients without SAMS had a significantly greater decrease in LDL-C (*p* = 0.04) suggests that SAMS may negatively impact statin effectiveness. This could be attributed to non-adherence to the statin regimen due to the adverse effects. However, this hypothesis was not supported by the adherence analysis, though this may be due to the adherence data availability being limited to only 30 patients in this study.

Several important limitations may have affected the ability to detect true associations. The retrospective chart review design inherently limits data, especially pertaining to medication adherence. Medication adherence is inconsistently documented in the electronic health record (EHR), necessitating reliance on prescription fill histories as surrogate measures. The authors’ institutional EHR medication fill history is sourced through a third-party platform with several limitations: (1) data availability extends only two years retrospectively, (2) fills paid out-of-pocket or through discount programs are not captured, (3) not all insurance plans report to the third-party platform, and (4) prescription fills do not necessarily correlate with medication pickup or actual consumption. Given these limitations, adherence information was available for only 30 of the included patients. The authors acknowledge that these constraints limit their ability to accurately assess true medication adherence, and they utilized all available data sources to provide the most comprehensive assessment possible within these limitations. This critical limitation likely contributed to insufficient statistical power to detect clinically meaningful differences, and adherence was thus analyzed in an exploratory manner. Type II error is a significant concern, and these negative findings should be interpreted cautiously in this context. The effect sizes for *SLCO1B1* function on statin effectiveness may be smaller than those observed for SAMS risk, requiring substantially larger sample sizes to detect with adequate power. Furthermore, the study population was predominantly White, which limits the generalizability of the findings to more diverse populations and may not reflect the full spectrum of *SLCO1B1* variant effects across different ethnic groups. Given the significant allele frequency differences of statin-related pharmacokinetic genes across major racial groups [17], the impact of SLCO1B1*5 on statin effectiveness underscores the importance of validating findings across diverse populations. Reports of statin-induced myopathy in certain racial groups suggest that population-level responses may be driven, at least in part, by population-specific variants [18]. In addition, differences in linkage disequilibrium patterns, co-inherited variants, and gene–environment interactions may further influence drug disposition and the risk of adverse events. These genetic factors can be compounded by socioeconomic and healthcare access disparities, which may affect adherence, prescribing patterns, and clinical outcomes. Therefore, cross-population studies are essential not only to confirm the clinical relevance of *SLCO1B1* variants but also to identify additional genetic, biological, and contextual determinants of statin response and safety.

The broad baseline and follow-up windows employed in this study reflect real-world clinical practice patterns, where patients present for care and follow-up at varying intervals based on individual circumstances, provider availability, and clinical need. While narrower temporal windows provide greater precision, they would compromise the external validity and generalizability of the findings to routine clinical practice. The authors implemented clinically relevant constraints to balance precision with real-world applicability. Baseline LDL-C measurements were restricted to within one year prior to statin initiation, as clinical practice typically relies on LDL-C values obtained within this timeframe to guide statin prescribing decisions. For follow-up assessment, LDL-C levels were required to be obtained at least six weeks post-initiation, consistent with recommendations from the American Association of Clinical Endocrinologists (AACE) and American College of Endocrinology (ACE) Guidelines for Management of Dyslipidemia and Prevention of Cardiovascular Disease [19]. These guidelines recommend testing at six-week intervals until treatment goals are achieved, followed by 6- to 12-month monitoring intervals thereafter. Through comprehensive chart review, the authors excluded patients with documented statin discontinuation prior to follow-up laboratory assessment. The observed average duration of statin therapy at first follow-up LDL-C assessment was 10 months, which while not ideal, reflects typical clinical monitoring practices. The authors acknowledge that follow-up time was not incorporated as a covariate in the primary analysis, which represents a limitation as variable treatment durations could influence observed LDL-C responses. However, since maximum LDL-C lowering effectiveness is typically achieved within 12 weeks of statin initiation [1], this effect is expected to have been captured by the mean follow-up timing of 10 months.

The exclusion of patients who had pharmacogenomic testing specifically ordered to guide statin selection due to past intolerance may have introduced bias by selectively omitting patients most likely to be influenced by *SLCO1B1* genotype, potentially diluting observable genotype–phenotype relationships. However, these patients would likely have had suboptimal adherence to their previous statin therapy due to adverse effects, which could confound the assessment of genetic effects on statin effectiveness. The exclusion of patients who initiated statin therapy after their pharmacogenomic results were available was necessary to minimize confounding, as knowledge of *SLCO1B1* phenotype could have influenced both statin selection and dosing decisions, making it impossible to assess the natural relationship between genotype and treatment outcomes.

An important pharmacogenomic limitation involves the absence of *ABCG2* genotyping from the commercial PGx panel most commonly utilized at the study institution. The *ABCG2* transporter plays a role in rosuvastatin transport and absorption, and genetic variation in this gene could confound the relationship between *SLCO1B1* and rosuvastatin response [2,20,21], which comprised a substantial portion of the study population. *CYP2C9*, which is the primary metabolizing enzyme of fluvastatin [22,23], was also not considered. However, this is not likely to significantly impact the results as only 1 out of the 213 included patients was prescribed fluvastatin.

It is worth noting that the impact of *SLCO1B1* variation on statin exposure varies across statins. Simvastatin acid is exclusively dependent on SLCO1B1 for hepatic uptake [24]. In contrast, atorvastatin and other statins utilize additional hepatic uptake transporters including SLCO1B3 and SLCO2B1, providing alternative pathways that may partially compensate when SLCO1B1 function is impaired [24]. Consequently, the effect of the *SLCO1B1* *5/*5 genotype, which confers poor transporter function, is most pronounced for simvastatin acid, leading to a marked increase in simvastatin exposure [24]. This pharmacokinetic distinction is pertinent to the present study cohort, where atorvastatin predominated (58.2%) versus simvastatin (15%), potentially explaining why lower than expected rates of statin ineffectiveness and intolerance among variant carriers were observed. These findings underscore the importance of considering specific statin type when evaluating the clinical utility of *SLCO1B1* genotyping.

While the *5 allele was the focus of the study’s genotyping approach, there are additional variants in *SLCO1B1* that are known to impair the transporter function, e.g., SLCO1B1*9: c.1463G>C (rs59502379) and *23: c. (rs373327528). These were not included in the commercial clinical assay that is commonly used by the study institution. However, this is unlikely to have a major impact on the results of this study since 97.2% of the study cohort was White, and the frequencies of these variants are very low (<0.1%) in those of European ancestry [25].

Future research should prioritize prospective cohort studies with larger, more diverse patient populations to provide adequate statistical power for detecting clinically meaningful effects. Improved adherence measurement methodologies would enhance the validity of effectiveness assessments. Multi-center studies spanning different health networks would improve generalizability and provide the sample sizes necessary for robust pharmacogenomic associations. A re-analysis of statin studies for which *SLCO1B1* sequencing data are available may also provide valuable insight into this variant (or others) and statin effectiveness. Additionally, comprehensive pharmacogenomic panels that include *ABCG2* and *CYP2C9* should be incorporated to account for the polygenic nature of statin response.

## 5. Conclusions

This study does not support a significant impact of *SLCO1B1* genetic variation on statin effectiveness, SAMS occurrence, or adherence. However, methodological limitations, particularly the small sample size and retrospective design, limit the definitive nature of these conclusions. While these findings do not demonstrate clinical utility of *SLCO1B1* testing for predicting statin effectiveness, definitive conclusions regarding the absence of genetic effects cannot be drawn. This study may have been underpowered to detect clinically relevant associations, particularly considering the relatively small cohort and unequal representation across *SLCO1B1* phenotype categories. The current evidence base remains insufficient to guide clinical decision-making using *SLCO1B1* variation as a biomarker for statin effectiveness. Despite these findings, the established role of *SLCO1B1* in SAMS risk prediction, as outlined in the 2022 CPIC guidelines [3], remains clinically relevant and should continue to inform prescribing decisions focused on safety optimization. Importantly, *SLCO1B1* variation is not a contraindication to statin therapy, and statin therapy remains recommended for patients in whom a statin is indicated regardless of *SLCO1B1* variation. Until larger prospective validation studies with enhanced adherence monitoring and comprehensive pharmacogenomic assessment definitively characterize the relationship between *SLCO1B1* function and statin therapeutic outcomes, clinicians should continue to rely on established clinical parameters and the recognized association between *SLCO1B1* and SAMS risk when making statin prescribing decisions.

## Figures and Tables

**Table 1:** Baseline characteristics.

Included patients, N	213
*SLCO1B1* Functional Status, n (%)	
Normal Function (*1/*1)	145 (68.1)
Decreased Function (*1/*5)	60 (28.2)
Poor Function (*5/*5)	8 (3.76)
*CYP3A4* Phenotypes, n (%)	
Normal Metabolizer (*1/*1, *1/*1A, *1A,*1A)	191 (89.6)
Intermediate Metabolizer (*1/*22, *22/*22)	21 (9.9)
Data Unavailable	1 (0.5)
*CYP3A5* Phenotypes, n (%)	
Non-expressor (*3/*3, *3/*6, *3/*7, *6/*6,*6/*7, *7/*7)	185 (86.9)
Expressor (*1/*1, *1/*3, *1/*6, *1/*7)	27 (12.6)
Data Unavailable	1 (0.5)
Sex, n (%)	
Female	124 (58.2)
Race, n (%)	
White	207 (97.2)
Age: Years, mean (±SD)	58 ± 12.4
BMI: kg/m^2^, mean (±SD)	34 ± 16.6
Current Smoker, n (%)	51 (23.9)
Diabetes, n (%)	50 (23.5)
eGFR < 60 mL/min/1.73m^2^, n (%)	10 (4.7)
Overall Baseline LDL-C: mg/dL, mean (±SD)	142 ± 35.8
Baseline LDL-C by *SLCO1B1* function: mg/dL, mean (±SD)	
Normal Function	141 ± 37.1
Decreased Function	141 ± 31.7
Poor Function	153 ± 41.0
Statin Indication, n (%)	
Primary Prevention	187 (87.8)
Secondary Prevention	26 (12.2)
Statin Medication, n (%)	
Atorvastatin	124 (58.2)
Simvastatin	32 (15.0)
Rosuvastatin	28 (13.1)
Pravastatin	23 (10.8)
Lovastatin	3 (1.4)
Pitavastatin	2 (0.9)
Fluvastatin	1 (0.5)
Statin Intensity, n (%)	
High Intensity	25 (11.7)
Moderate Intensity	155 (72.8)
Low Intensity	33 (15.5)
Concurrent Strong *CYP3A4* Inducer, n (%)	
Carbamazepine	1 (0.5)
Concurrent Moderate *CYP3A4* Inhibitor, n (%)	
Diltiazem	3 (1.4)
Verapamil	1 (0.5)

N = number of patients in the study population; n = number of patients per group; SD = standard deviation; BMI = body mass index; eGFR = estimated glomerular filtration rate; LDL-C = low-density lipoprotein cholesterol.

**Table 2:** Effect of statin intensity on mean change in LDL-C by *SLCO1B1* variation.

*SLCO1B1* Function	Mean Change in LDL-C (%)				
	Overall	*p*-Value	Statin Intensity: High	Statin Intensity: Moderate	Statin Intensity: Low
Normal (n = 145)	−29.8	0.24	−40	−31.1	−15.5
Decreased (n = 60)	−36.1		−45.1	−38.9	−18.9
Poor (n = 8)	−35.6		−52.7	−38.6	−0.9
***p*-value**			0.8	0.13	0.86
**Overall Mean Change in LDL-C (%) by Statin Intensity**					
Statin Intensity: High (n = 25)				−42.1	
Statin Intensity: Moderate (n = 155)				−33.4	
Statin Intensity: Low (n = 33)				−16.2	

*SLCO1B1* variation is defined as *SLCO1B1* function: normal, decreased, or poor. *p*-values calculated using one-way ANOVA. n = number of patients; LDL-C = low-density lipoprotein cholesterol.

**Table 3:** Occurrence of SAMS based on *SLCO1B1* variation.

Overall SAMS Occurrence, n (%)			
29 (13.6)			
*SLCO1B1* Function	SAMS Occurrence		*p*-Value
	Yes, n (%)	No, n (%)	
Normal	19 (8.9)	126 (59.2)	0.24
Decreased	10 (4.7)	50 (23.5)	
Poor	0 (0)	8 (3.8)	

*SLCO1B1* variation is defined as *SLCO1B1* function: normal, decreased, or poor. *p*-value calculated using chi-square test of independence. n = number of patients; SAMS = statin-associated muscle symptoms.

**Table 4:** Statin adherence by *SLCO1B1* variation.

Overall Adherent, n (%)			
22 (73.3)			
*SLCO1B1* Function	Adherent, n (%)	Not Adherent, n (%)	*p*-Value
Normal	15 (50)	5 (16.7)	1.00
Decreased	5 (16.7)	3 (10)	
Poor	2 (6.6)	0 (0)	

*SLCO1B1* variation is defined as *SLCO1B1* function: normal, decreased, or poor. *p*-value calculated using Fisher’s exact test. n = number of patients.

**Table 5:** Effect of SAMS on average change in LDL-C and adherence.

SAMS	Average Change in LDL-C (%)	
	Adherent (n = 22)	Not Adherent (n = 5)
Yes (n = 2)	−2.19	−9.9
No (n = 28)	−40.5	−12
***p*-value**	**0.04**	0.95

*p*-values calculated using one-way ANOVA. n = number of patients; SAMS = statin-associated muscle symptoms.

**Table 6:** Effect of SAMS on adherence.

SAMS	Adherent (n)	Not Adherent (n)	*p*-Value
Yes	1	1	0.44
No	21	7	

*p*-value calculated using Fisher’s exact test. n = number of patients; SAMS = statin-associated muscle symptoms.

## Data Availability

The raw data supporting the conclusions of this article will be made available by the authors on request.

## Abbreviations

The following abbreviations are used in this manuscript:

ALT	Alanine transaminase
ANOVA	Analysis of variance
ASCVD	Atherosclerotic cardiovascular disease
BMI	Body mass index
CI	Confidence interval
CKD	Chronic kidney disease
CPIC	Clinical Pharmacogenetics Implementation Consortium
CTT	Cholesterol Treatment Trialists
CVD	Cardiovascular disease
eGFR	Estimated glomerular filtration rate
EHR	Electronic Health Record
HMG-CoA	Hydroxymethylglutaryl-coenzyme A
LDL-C	Low-density lipoprotein cholesterol
PCSK9i	Proprotein convertase subtilisin/kexin type 9 inhibitors
PDC	Proportion of days covered
PGx	Pharmacogenomic/pharmacogenomics
PharmVar	Pharmacogene Variation Consortium
SAMS	Statin-associated muscle symptoms
SLCO1B1	Solute carrier organic anion transporter family member 1B1
